# Concentration of *Plasmodium falciparum* gametocytes in whole blood samples by magnetic cell sorting enhances parasite infection rates in mosquito feeding assays

**DOI:** 10.1186/s12936-017-1959-9

**Published:** 2017-08-05

**Authors:** Isaie J. Reuling, Will J. R. Stone, Marga van de Vegte-Bolmer, Geert-Jan van Gemert, Rianne Siebelink-Stoter, Wouter Graumans, Kjerstin Lanke, Teun Bousema, Robert W. Sauerwein

**Affiliations:** Department of Medical Microbiology, Radboud University Medical Center, Geert Grooteplein 28, Microbiology 268, 6500 HB Nijmegen, The Netherlands

**Keywords:** Malaria, Gametocytes, Mosquito-feeding assay, MACS

## Abstract

**Background:**

Mosquito-feeding assays are important tools to guide the development and support the evaluation of transmission-blocking interventions. These functional bioassays measure the sporogonic development of gametocytes in blood-fed mosquitoes. Measuring the infectivity of low gametocyte densities has become increasingly important in malaria elimination scenarios. This will pose challenges to the sensitivity and throughput of existing mosquito-feeding assay protocols. Here, different gametocyte concentration methods of blood samples were explored to optimize conditions for detection of positive mosquito infections.

**Methods:**

Mature gametocytes of *Plasmodium falciparum* were diluted into whole blood samples of malaria-naïve volunteers. Standard centrifugation, Percoll gradient, magnetic cell sorting (MACS) enrichment were compared using starting blood volumes larger than the control (direct) feed.

**Results:**

MACS gametocyte enrichment resulted in the highest infection intensity with statistically significant increases in mean oocyst density in 2 of 3 experiments (*p* = 0.0003; *p* ≤ 0.0001; *p* = 0.2348). The Percoll gradient and standard centrifugation procedures resulted in variable infectivity. A significant increase in the proportion of infected mosquitoes and oocyst density was found when larger volumes of gametocyte-infected blood were used with the MACS procedure.

**Conclusions:**

The current study demonstrates that concentration methods of *P. falciparum* gametocyte-infected whole blood samples can enhance transmission in mosquito-feeding assays. Gametocyte purification by MACS was the most efficient method, allowing the assessment of gametocyte infectivity in low-density gametocyte infections, as can be expected in natural or experimental conditions.

**Electronic supplementary material:**

The online version of this article (doi:10.1186/s12936-017-1959-9) contains supplementary material, which is available to authorized users.

## Background

Malaria transmission is mediated by sexual stage parasites (gametocytes) that are generated at low frequency from their asexual progenitors. Mature gametocytes are ingested by mosquitoes, activating inside the mosquito gut to form micro- (male) and macro- (female) gametes. Gametes sexually reproduce to form oocysts, inside which infectious sporozoites develop that make the mosquito infectious to humans once they reach the mosquito salivary glands.

Transmission-reducing interventions form essential components of global efforts to contain anti-malarial resistance [1] and eliminate malaria [2]. Such interventions will lower malaria transmission by reducing gametocyte production and carriage, or by interfering with sporogonic development in the mosquito. Mosquito-feeding assays are important tools to guide the development and support the evaluation of transmission-blocking interventions. These functional bioassays measure the transmission of gametocytes to mosquitoes, by allowing mosquitoes to feed either on blood from infected individuals (the direct skin or direct membrane-feeding assay) [3] or cultured gametocytes (the standard membrane-feeding assay; SMFA), before assessing the infection rate in the mosquitoes by oocyst or sporozoite detection [4]. Higher densities of gametocytes are associated with higher mosquito infection rates [4], but mosquito infections may occur at very low gametocyte densities [5, 6]. The stochastic nature of mosquito infections at low oocyst densities makes it difficult to judge whether non-infectiousness of gametocyte-positive blood samples arises because: (i) gametocytes are too low in density [7]; (ii) too few mosquitoes were examined; or (iii) other factors influencing gametocyte infectiousness (e.g. parasite factors [8, 9] such as maturity [10, 11] or sex ratio [12, 13]. or extrinsic factors such as human or mosquito immunity [14–16]).

Measuring the viability of low gametocyte densities becomes increasingly important in malaria elimination scenarios as low density infections with gametocytes may be very common [17]. Under such conditions sporadic mosquito transmission events may be highly relevant for control efforts but will require a large number of mosquitoes to be examined.

As field studies are subject to many uncontrolled and likely confounding parameters, a controlled human malaria infection (CHMI) model for transmission from man to mosquito would be a great asset for the evaluation of (transmission-blocking) drugs or vaccines [18, 19], (Reuling et al. pers. Comm..). Here, different gametocyte concentration methods in blood samples were explored with the aim to optimize conditions for detection of positive mosquito infections.

## Methods

### In vitro gametocyte culture

A semi-automated suspension culture was used for the gametocyte culture with slight modification of descriptions by Ponnudurai et al. [20]. Mature gametocytes of *Plasmodium falciparum* (NF54 strain) were cultured in a ‘shaker’ flask for periods of 16 days at 37 °C under 4% C0_2_, 3% O_2_, 93% N_2_ continuous gas flow. Parasites were cultured in RPMI 1640 with l-glutamine (Life Technologies), supplemented with 5.94 g/L HEPES (BDH Biochemical), 50 mg/L hypoxanthine (Sigma Aldrich), and 50 mM of *N*-acetyl glucosamine (Sigma Aldrich) to treat asexual parasites from day 8. Culture medium was changed twice daily, with an initial 4–5% haematocrit.

### Membrane-feeding assays

Cultured gametocytes were diluted into whole blood samples. Venous blood of malaria-naïve volunteers was drawn into heparin-containing tubes, and kept warm in a 37 °C heat-block. Cultured *P. falciparum* gametocytes were added to the whole blood at concentrations between 300 and 1000 gametocytes/µL (in standard experiments). Gametocytes concentrated by the various purification methods were all reconstituted in 400 µL of blood, of which 300 µL was transferred to an artificial membrane feeder and offered for exactly 15 min to a cage of 50, 1–3-day old *Anopheles stephensi* mosquitoes. The remaining 100 µL of blood was stored in RNAprotect Cell Reagent (Qiagen, Hilden, Germany). Control mosquitoes were fed the gametocyte-enriched whole blood directly (without concentration). Blood-fed mosquitoes were kept on glucose at 26 °C and 70–80% humidity; on day 7 post infection the mid-guts of 20 mosquitoes in each feed group were removed by dissection, stained with mercurochrome, and examined for the presence of oocysts by microscopy.

### Gametocyte concentration

All procedures were conducted at 37 °C and samples were kept at this temperature prior to feeding to prevent early gametocyte activation. Three purification methods were compared by using starting blood volumes 5 times higher than the control (direct) feed, to assess their suitability to retain or improve infectivity in the SMFA. Because only 300 µL of the 400 µL concentrated feed material was fed to mosquitoes, the remainder being used for gametocyte quantification, the SMFA sample contained 75% of the total gametocyte content of purification output (equivalent to a 3.75-fold higher start volume than the control).

### Standard centrifugation

Gametocytes were separated from uninfected erythrocytes and asexual parasites by standard centrifugation causing gametocytes to form a layer on top of the erythrocytes. Gametocyte-infected whole blood was spun down at 2000 RPM at 37 °C for 5 min. Two-hundred µL of the top-layer of the pellet of erythrocytes was added to 200 µL of plasma to adjust the haematocrit level (1:1 erythrocyte/plasma ratio).

### Percoll gradient separation

Gametocytes in the infected whole blood sample were concentrated by a 63% Percoll density gradient (Percoll, GE, USA) centrifugation step by centrifugation at 2000 RPM (3 min acceleration; 3 min break) at 37 °C for 20 min [21, 22]. The interface containing gametocytes was separated using a blunt needle, and washed twice with warm incomplete medium (RPMI 1640 parasite medium without 10% serum) at 2000 RPM for 5 min (1 min acceleration; 1 min break). The supernatant was removed leaving 50 µL, in which the pellet was re-suspended and mixed with 350 µL of uninfected whole blood.

### Magnetic cell sorting (MACS) for gametocyte enrichment

Gametocytes in the infected whole blood sample were concentrated by magnetic cell sorting (MACS) using a QuadroMACS™ separator and LS MACS columns (MiltenyiBiotech, UK) as described previously, with slight modifications [23, 24]. A 21-gauge (21G) needle (0.8 × 50 mm) was attached to the column. After equilibration with 1 mL of warm incomplete medium, gametocyte-infected whole blood was added and the column was washed with 3 mL of warm incomplete medium. Next, the column was then removed from the magnet, and placed in a 15-mL centrifuge tube and 1 mL of warm (37 °C) incomplete medium was added to the column. When the flow ceased, another 1 mL of medium was added to the column, and gently pressed through the column with the corresponding plunger. The gametocyte-enriched suspension was spun down in a 37 °C centrifuge for 10 min at 2000 RPM. The supernatant was removed leaving 50 µL, in which the pellet was re-suspended. 350 µL of uninfected whole blood was added to the pellet and kept at 37 °C until use. The entire MACS procedure was carried out in a heated cabinet incubator.

### Quantitative reverse transcriptase polymerase chain reaction

Quantitative reverse transcription PCR (qRT-PCR) was performed targeting Pfs25 mRNA that is specific to female gametocytes [40]. One-hundred µL of the samples (gametocyte enriched or control) were added to 400 µL of RNAprotect Cell Reagent (Qiagen, Hilden, Germany) for automated extraction and qRT-PCR as described previously [25] with two modifications: 2 µL cDNA was used in the 20 µL final reaction mix and primer concentration was reduced to 225 nM.

### Statistical analysis

Statistical analysis was performed using GraphPad Prism software version 5 (GraphPad Software Inc., California, USA). Differences in oocyst intensity between feeds were determined by the Mann–Whitney U test, and Fisher’s exact test was used to test for differences in oocyst prevalence.

## Results

### Comparison of gametocyte concentration methods

**Fig. 1:**
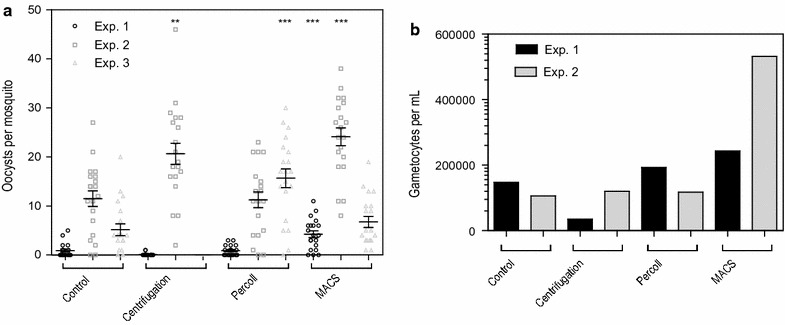
Infectivity of gametocyte-infected whole blood samples after different concentration methods. **a** The starting material of gametocyte-infected whole blood was 300 µL for the controls, which was fed to mosquitoes directly. For all non-control feeds, starting material was 1500 µL of the same gametocyte-infected whole blood. Gametocytes from this starting material were concentrated, eluted and reconstituted in 400 µL of uninfected blood, from which 300 µL was fed to the mosquitoes.* Vertical bars* indicate mean with SEM. **p < 0.01, ***p < 0.001. **b** Gametocyte density per sample from feeding material measured by Pfs25 qRT-PCR from experiments 1 and 2

Three independent experiments were conducted to compare oocyst intensities between standard centrifugation (Centrifugation), Percoll gradient separation (Percoll), and magnetic cell sorting (MACS) (Fig. 1). MACS gametocyte enrichment resulted in statistically significant increases, relative to control, in mean oocyst density in 2 of 3 experiments, and numerically higher in the third experiment (*p* = 0.0003; *p* ≤ 0.0001; *p* = 0.2348) (Fig. 1a, Table 1). The effect of MACS enrichment on infection prevalence (the proportion of infected mosquitoes) was statistically significant in only 1 of 3 experiments (*p* = 0.0187; *p* = 0.4872; *p* = 0.106). The Percoll gradient and standard centrifugation procedures showed variable infectivity results (Fig. 1a, Table 1). The mean oocyst density ranged from 0.9–11.5, 0.1–20.7, 0.9–15.7, and 4.3–24.1, for the control, standard centrifugation, Percoll, and MACS, respectively. MACS gametocyte enrichment showed a 31, 378 and 110% increase in mean oocysts relative to control. Percoll gradient gave the highest oocyst density in one experiment with a 202% increase in mean oocysts relative to control, compared to MACS with 31%. However, the other two experiments conducted with Percoll gradient did not increase oocyst density relative to control, while the use of MACS consistently resulted in an increased oocyst density. Gametocyte density of mosquito feed material as determined by Pfs25 qRT-PCR from experiments 1 and 2 are shown in Fig. 1b. A procedural error occurred during the qRT-PCR sampling process of experiment 3, resulting in loss of material. Whilst these findings are based on only two experiments, and do not reflect the proportional increase in gametocyte numbers that was expected based on input volume, they suggest a pronounced increase in gametocyte numbers by MACS.

**Table 1:** Details of the comparison of concentration methods. *n/N* = Infected mosquitoes/total number of mosquitoes dissected

Experiment	Protocol	Blood volume (µL)^a^	Total oocysts	Infected mosq. n/N (%)	*p* value*	Mean oocysts (range)	% change in mean oocyst to control	*p* value*
1	Control	300	18	9/20 (45%)	–	0.9 (0–5)	–	–
	Centrifugation	1500	2	2/20 (10%)	0.031	0.1 (0–1)	−88.9	0.0112
	Percoll	1500	18	11/20 (55%)	0.7524	0.9 (0–3)	0	0.6185
	MACS	1500	85	17/20 (85%)	*0.0187*	4.3 (0–11)	377.8	*0.0003*
2	Control	300	230	18/20 (90%)	–	11.5 (0–27)	–	–
	Centrifugation	1500	413	20/20 (100%)	0.4872	20.7 (2–46)	80	*0.0011*
	Percoll	1500	225	18/20 (90%)	1	11.3 (0–23)	−1.7	0.8494
	MACS	1500	482	20/20 (100%)	0.4872	24.1 (8–38)	109.6	<*0.0001*
3	Control	300	103	18/20 (80%)	–	5.2 (0–20)	–	–
	Centrifugation	1500	0	0/20 (0%)	<0.0001	0 (0)	−100	<0.0001
	Percoll	1500	313	19/20 (95%)	0.3416	15.7 (0–30)	201.9	*0.0002*
	MACS	1500	135	20/20 (100%)	0.106	6.8 (1–14)	30.8	0.2348

* Significance compared to paired control

^a^Before concentration; blood volume in feeder 300 µL

### The effect of larger blood sample aliquots on gametocyte infectivity by MACS

**Fig. 2:**
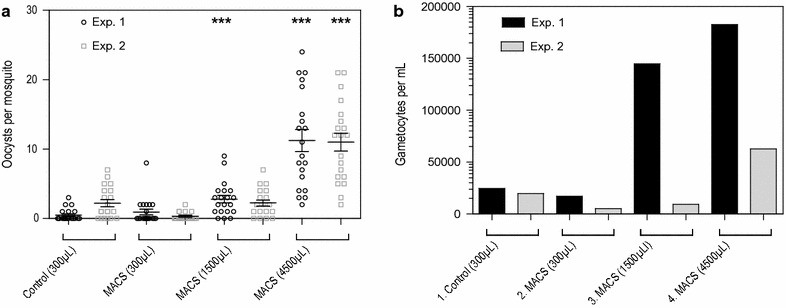
The effect blood sample size on gametocyte concentration and infectivity by MACS. **a** The starting material of gametocyte-infected whole blood was 300 µL for the controls, which was fed to mosquitoes directly. For the MACS, starting material was 300, 1500 and 4500 µL, respectively. Gametocytes from this starting material were concentrated, eluted and reconstituted in 400 µL of uninfected blood, from which 300 µL was fed to the mosquitoes.* Vertical bars* indicate mean with SEM. ***p < 0.001. **b** Gametocyte density per sample from feeding material measured by Pfs25 qRT-PCR

Next, the possibility of using larger starting volumes of infected whole blood by MACS for gametocyte enrichment was examined. The infectiousness of gametocytes purified from 300, 1500 and 4500 µL of gametocyte-infected blood was assessed in two separate experiments (Fig. 2a). The control direct feed with 300 µL of gametocyte-infected whole blood resulted in a mean of 0.5 (range 0–3) and 2.2 (range 1–7) oocysts per mosquito in two separate experiments. MACS gametocyte enrichment conducted with 300 µL of purified blood gave respective mean oocyst densities of 0.95 (range 0–8), and 0.35 (range 0–2) in the two independent experiments; when using 1500 µL, mean oocyst density increased to 2.8 (range 0–9) and 2.25 (range 0–7). Finally, using 4500 µL of blood increased the mean number of oocysts to 11.1 (range 11–11.25), reflecting a 5- to 22.5-fold difference, compared to the controls. Gametocyte density of all mosquito feed material as determined by Pfs25 qRT-PCR is shown in Fig. 2b.

### Enhancing infectivity of low gametocyte densities by MACS

**Fig. 3:**
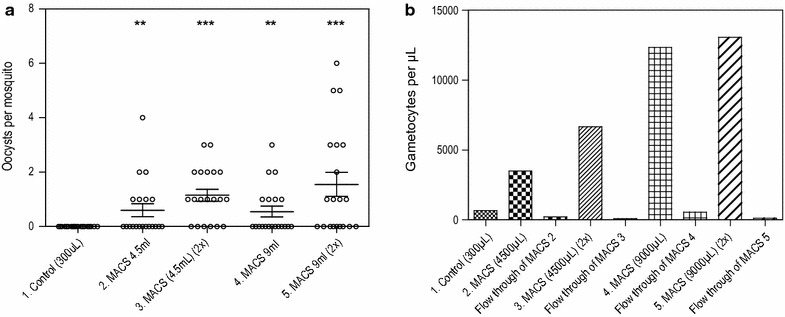
Concentration and infectivity of low gametocyte densities after MACS. **a** *1* 300 µL of gametocyte-infected whole blood from starting material, fed directly (control). *2* 4500 µL of gametocyte-infected whole blood from starting material added once over MACS column, eluted and reconstituted in 400 µL of uninfected blood, from which 300 µL was fed. *3* 4500 µL of gametocyte-infected whole blood from starting material run twice over MACS column, eluted and reconstituted in 400 µL of uninfected blood, from which 300 µL was fed. *4* 9000 µL of gametocyte-infected whole blood from starting material added once over MACS column, eluted and reconstituted in 400 µL of uninfected blood, from which 300 µL was fed. *5* 9000 µL of gametocyte-infected whole blood from starting material run twice over MACS column, eluted and reconstituted in 400 µL of uninfected blood, from which 300 µL was fed. *Vertical bars* indicate mean with SEM. **p < 0.01, ***p < 0.001. ** b** Gametocyte density per sample from feeding material measured by Pfs25 qRT-PCR. Flow through of MACS represents the loss of gametocytes after MACS purification

MACS gametocyte enrichment at low gametocyte densities was studied with the gametocyte density in culture material of ~400 gametocytes/µL and in titration series from 1 to 6400 gametocytes/µL. The gametocyte content of the 4500 and 9000 µL start volumes was thus equivalent to 11.25- and 22.5-fold higher start volumes than the control. While the control feed showed no infection of mosquitoes, up to 70% of mosquitoes were infected using the 4500 and 9000 µL samples (Fig. 3a). The total number of gametocytes per sample after purification was higher when a larger volume of blood was used and when the blood was run twice over the MACS (Fig. 3b). The material collected from the flow-through of the MACS prior to its removal from the magnet was tested in the qRT-PCR. The flow-through contained approximately 5–9% of the total gametocyte content of the start material that was not retained by the magnet. Follow-on experiments indicated that this loss could be reduced to ~1% by running the blood twice over the MACS, thereby maximizing the likelihood of successful adherence of gametocytes to the magnetic column.

**Fig. 4:**
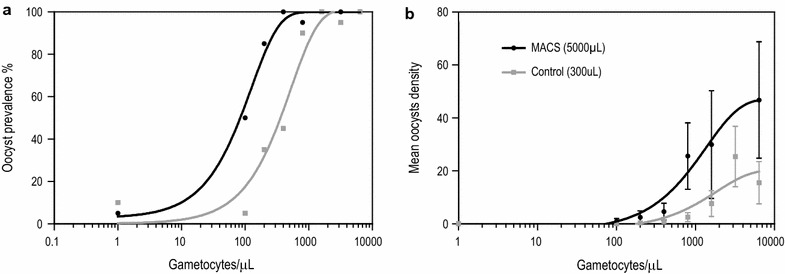
Infectivity of different gametocyte densities after MACS versus control in a titration experiment. **a** Percentage of infected mosquitoes after titration experiment with MACS versus controls. **b** Number of oocysts per mosquito after titration experiment with MACS versus controls. *Vertical bars* indicate mean with SEM

Gametocytes were titrated to assess mosquito infection rates after MACS at different gametocyte densities as determined by microscopy (Fig. 4a, b). The starting concentration and subsequent dilutions were either fed directly to mosquitoes or used for MACS gametocyte enrichment. Plotted are infection rates relative to the original gametocyte density. Control feeds used 300 µL of gametocyte-infected whole blood versus 5000 µL in the MACS gametocyte-enrichment feeds. A significant increase in the proportion of infected mosquitoes and oocyst density was found when using the MACS with the same gametocyte density, but a higher volume of gametocyte-infected blood. These increases in transmission resulted in a lower minimum gametocyte density to successfully measure transmission to mosquitoes.

Gametocyte densities were positively associated with infection rates (*r* = 0.6037, *p* ≤ 0.0001) and mean oocyst numbers (*r* = 0.5968, *p* ≤ 0.0001) in the general experiments as shown in Additional file 1: Figure S1.

## Discussion

The current study successfully demonstrates that different concentration methods of *P. falciparum* gametocyte-infected whole blood samples enhance transmission in mosquito-feeding assays. The MACS gametocyte enrichment method showed an increase in oocyst intensity compared to control in all replicates. The MACS has been used previously for gametocyte synchronization and gametocyte detection in field studies [26]. This study shows that gametocytes remain infective after passage over a MACS column with densities as low as 1–100 gametocytes/µL (Table 1).

Assessing the infectivity of low-density gametocytes is relevant for malaria control for areas with high prevalence of low-density infections [17, 27]. The stochastic nature of mosquito infections at low gametocyte densities complicates assessments of their transmissibility. An impractical number of mosquitoes needs to be examined to rule out infectivity with certainty. Whilst mosquito infection rates (and the intensity of infection in these mosquitoes) increase with increasing gametocyte density [28] a non-negligible fraction of high-density gametocyte infections appears to be sterile and unable to infect mosquitoes [3, 29]. It is increasingly recognized that the presence or density alone does not equal their infectiousness and direct assessments of the viability of gametocytes are essential [30, 31]. In epidemiological and intervention studies, it may thus be of relevance to determine whether gametocytes are capable of transmission regardless of their density. Sensitive mosquito feeding assays may be of particular relevance to study the dynamics of gametocyte infectivity in natural infections. Experimental infections suggests that infectivity may peak early in infections and gametocytes that are observed later in infections may be comparatively less infectious or non-infectious [32]. If the same applies to natural infections, this would be highly relevant since some gametocyte infections may thus be considered less important for malaria transmission. Similarly, clinical trials that specifically aim to induce gametocytes in volunteers may require an operationally attractive approach to enrich for gametocytes to increase the chances of successful mosquito infection and demonstrate the generation of viable gametocytes in these trials.

The MACS gametocyte enrichment method is simple, rapid and has been used extensively to concentrate gametocytes for purposes other than mosquito infection [23]. In the current experiments, MACS gave repeatedly high infection intensities compared to control and was, therefore, studied in more detail to test its ability to concentrate gametocytes from input material with low gametocyte concentrations. For this purpose, saturation of columns forms a concern in MACS procedures. The current study shows that the saturation point of the MACS was not reached when using a relatively large volume of 9000 µL of blood with an estimated number of 9,000,000 gametocytes (at 1000/µL). As previously shown, a reduction in flow rate through the MACS column could enhance the yield of *Plasmodium berghei* ookinetes [33], but no significant difference was observed when the flow rate was reduced by ~1.5 times, with a 23G needle compared to the 21G needle (Additional file 2: Figure S2). Nevertheless, further alteration of the flow rate may increase the efficiency of first pass column binding, and should therefore be investigated in future studies.

Previously, it has been reported that magnetic fields may affect parasite viability by compromising the growth of asexual *P. falciparum* forms [34]. This may relate to inhibition of polymerization of haem into haemozoin, and organelle damage due to the oscillation of haemozoin. In the current study, the magnetic field induced by the MACS appears to have limited impact on gametocyte fitness. Furthermore, no increase in infectivity was observed when concentrated gametocytes after MACS are brought back into culture for 24 h, whilst the risk of contamination was considerable (unpublished findings). In addition, handling time, stress and temperature drops during sample transfer may also impact gametocyte infectivity. In the current study these factors appeared to have limited impact on gametocyte fitness and infectivity and the advantages of gametocyte concentration outweighed their potential detrimental effects.

No formal optimization of alternative gametocyte-enrichment protocols was undertaken in this study. The current experiments therefore do not preclude that assay optimizations may increase transmission success of the tested enrichment procedures. Furthermore, the number of replicates was limited in the current study and the inevitable variation in transmission outcomes in standard membrane feeding assays with cultured gametocytes [35] warrants caution when comparing feeders within or between experiments. In addition, the lower efficiency of Percoll and standard centrifugation in the other experiments are most likely due to the low enrichment of gametocytes as reflected by the PCR results. When considering the other concentration methods evaluated, the standard centrifugation was indeed attractive because of its simplicity. However, the absence of an unequivocal visual cell layer made it difficult to reproducibly obtain a well-defined layer of gametocytes. An additional potential disadvantage of density-dependent centrifugation is that it is unable to separate gametocytes from host white blood cells [21, 22]. As host white blood cells can interfere with the success rate of transmission in membrane-feeding assays, such cell contamination is undesirable and will require another step of filtration [36–38]. Trang et al. shows that the MACS purification removes approximately 94% of the white blood cells, in contrast to 0% white blood cell removal after Percoll gradient, or other density gradient separation such as Nycodenz [39].

## Conclusions

The current study demonstrates that gametocyte purification by MACS concentrates gametocytes present at low density in whole blood samples whilst retaining their infectivity in membrane-feeding assays. Whilst current conclusions were based on a limited number of replicates, they formed the evidence base for the selection of a gametocyte enrichment method for a controlled human malaria infection study (CHMI) that aimed to induce (low-densities) gametocytes. In general, the methodology presented here may facilitate the use of blood samples from naturally or experimentally infected individuals to evaluate human host infectivity and malaria transmission-blocking interventions.

## Additional files

**Additional file 1: Figure S1.** Association between gametocyte densities and oocyst—prevalence and density. A. Overview of gametocyte densities and infection rates of experiments of Figs. 1, 2, 3, 4 and Additional file 2: Figure S2. B. Overview of gametocyte densities and mean oocysts of experiments of Figs. 1, 2, 3, 4 and Additional file 2: Figure S2.

**Additional file 2: Figure S2.** Effect of different flow-rate MACS on gametocyte infectivity. Number of oocysts per mosquito after membrane-feeding assay (MIDI cages). *1* 600 µL of gametocyte-infected whole blood from starting material, fed directly. *2* 9000 µL of gametocyte-infected whole blood from starting material added once over MACS column with 23G needle, eluted and reconstituted in 600 µL of uninfected blood before feed. *3* 9000 µL of gametocyte-infected whole blood from starting material added once over MACS column with 21G needle, eluted and reconstituted in 600 µL of uninfected blood before feed. Vertical bars indicate mean with SEM.

## Abbreviations

SMFA: standard membrane-feeding assay
CHMI: controlled human malaria infection
MACS: magnetic cell sorting
qRT-PCR: quantitative reverse transcription PCR
