# Supplementary material for: Concentration of Plasmodium falciparum gametocytes in whole blood samples by magnetic cell sorting enhances parasite infection rates in mosquito feeding assays
Source: Malar J. 2017 Aug 5;16:315. doi: 10.1186/s12936-017-1959-9 (PMC5545093; doi:10.1186/s12936-017-1959-9)
Supplement: Supplementary file 1 [file 12936_2017_1959_MOESM1_ESM.docx]

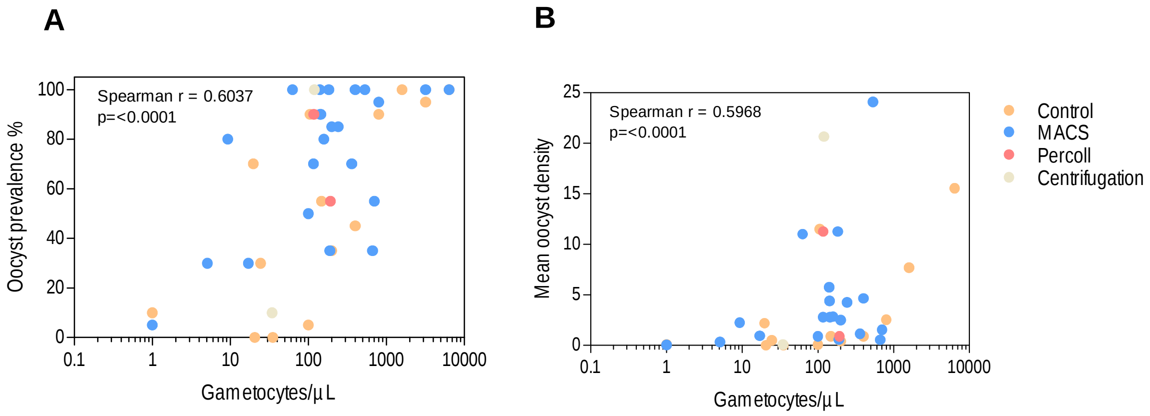

A
Spearman r = 0.6037
p=<0.0001
Oocyst prevalence %
100
80
60
40
20
0
0.1
1
10
100
1000
10000
Gametocytes/μL
B
Spearman r = 0.5968
p=<0.0001
Mean oocyst density
25
20
15
10
5
0
0.1
1
10
100
1000
10000
Gametocytes/μL
Control
MACS
Percoll
Centrifugation
