# Supplementary material for: Concentration of Plasmodium falciparum gametocytes in whole blood samples by magnetic cell sorting enhances parasite infection rates in mosquito feeding assays
Source: Malar J. 2017 Aug 5;16:315. doi: 10.1186/s12936-017-1959-9 (PMC5545093; doi:10.1186/s12936-017-1959-9)
Supplement: Supplementary file 2 [file 12936_2017_1959_MOESM2_ESM.docx]

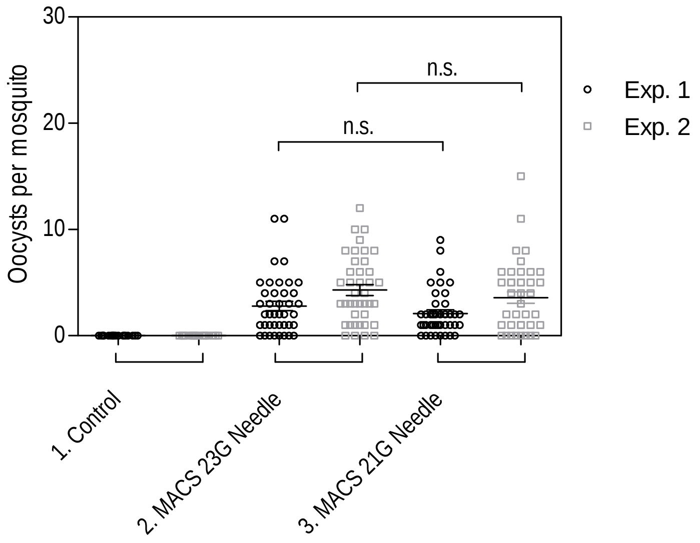
Oocysts per mosquito
30
20
10
0
n.s.
n.s.
Exp. 1
Exp. 2
1. Control
2. MACS 23G Needle
3. MACS 21G Needle
